# Highly Multiplexed Single‐Cell Protein Profiling with Large‐Scale Convertible DNA‐Antibody Barcoded Arrays

**DOI:** 10.1002/advs.201800672

**Published:** 2018-08-02

**Authors:** Peng Zhao, Sirsendu Bhowmick, Jianchao Yu, Jun Wang

**Affiliations:** ^1^ Multiplex Biotechnology Laboratory Department of Chemistry University at Albany State University of New York Albany NY 12222 USA; ^2^ Cancer Research Center University at Albany State University of New York Rensselaer NY 12144 USA

**Keywords:** barcoded arrays, DNA encoding, multiplex, protein detection, single‐cell measurements

## Abstract

Highly multiplexed detection of proteins secreted by single cells is always challenging. Herein, a multiplexed in situ tagging technique based on single‐stranded DNA encoded microbead arrays and multicolor successive imaging for assaying single‐cell secreted proteins with high throughput and high sensitivity is presented. This technology is demonstrated to be capable of increasing the multiplexity exponentially. Upon integration with polydimethylsiloxane microwells, this platform is applied to detect ten immune effector proteins from differentiated single macrophages stimulated with lipopolysaccharide. Significant heterogeneity is observed when the derived human primary macrophages are analyzed. This versatile technology is expected to open new opportunities in systems biology, immune regulation studies, signaling analysis, and molecular diagnostics.

## 1 Introduction

Cellular diversity is an intrinsic trait of any multicellular system. Despite extensive study of cells, their complexity still poses major challenges to the identification of key cellular subsets and targets that are responsible for human diseases. As cells from the same source are usually only varied epigenetically and functionally, single‐cell functional proteome has emerged as a research field of intense interest owing to the significant role proteins play in cell type identification, signaling transduction, proliferation and apoptosis, transcription regulation, inflammation, and cell communication.^1^ The proteomic tools present a highly promising tool for variant applications in the fields of systems biology, pathology, cell biology, and clinical diagnostics.^2^, ^3^ The key power of such techniques comes from the synergistic combination of single‐cell measurements and quantitative detection of molecular targets.

Among all functional proteins, the proteins secreted from single cells are particularly difficult to analyze, they are not confined with the cell's membrane boundary and are accumulated dynamically in the extracellular environment, and the spectra of secreted proteins are varied dramatically between specialized cells.^4^ Recently, microfluidic‐based methods have drawn increasing attention recently as it can circumvent costly instruments, integrate multiple generic functions into one system, and only need extremely small amount of samples without much consumption of expensive biochemical reagents.^5^

One of the common limitations of conventional microfluidic‐based single‐cell protein assays is low multiplexity, which is often linked to fluorescence spectrum overlap. To overcome this hurdle, integration of microarrays into single‐cell microchips conveniently addresses that limitation by achieving multiplexity as high as 42.^6^ However, this method still does not overcome the limitation of spectrum overlap, and patterning barcode arrays with a pitch < 10 µm in large‐scale is technically challenging. Recently, a few cyclic approaches have been developed to improve the multiplexity, but they have not yet been capable of multiplexed detection of proteins with single‐cell resolution.^7^, ^8^, ^9^

Herein, we developed an easy‐to‐follow and reliable multiplexed in situ tagging (MIST) strategy potentially assaying tens to hundreds of molecular targets in single cells with high throughput and high sensitivity. We achieve this goal by integrating a multicolor, multicycle molecular profiling technique with barcoded microbead antibody arrays and a DNA encoded antibody library. To demonstrate the concept and feasibility, 20 single‐stranded DNA (ssDNA) are used to encode each particle of a DNA array, and the decoding only needs three fluorescent colors with three successive bleaching and imaging cycles. After conversion of the DNA arrays into barcoded antibody arrays, they were applied to THP‐1 monocyte derived macrophages and human primary macrophages to quantitatively measure ten proteins secreted by single cells. Our technology is robust, inexpensive, reproducible, and requiring minimal instruments to detect the signal. Meanwhile, its multiplexity (*M^N^*) can reach an unprecedented level simply by expanding the color types (M) used for each staining cycle or imaging round number (N).

## 2 Results and Discussion

### 2.1 Overview of MIST Strategy for Single‐Cell Proteins Detection

**Figure 1:**
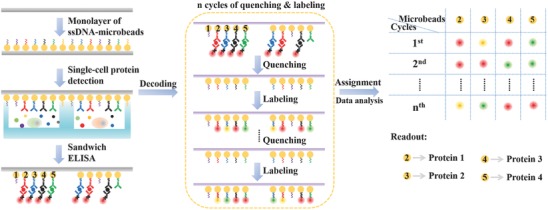
Scheme of the whole procedure for multiplexed single‐cell protein detection and decoding on the encoded MIST arrays. The location of each MIST array element (microbead) is recorded (labeled as 1, 2, .., 5) where some elements are used to detect secreted cytokines. Each microbead only carries a specific ssDNA sequence and detect only one species of protein. Quenching process is to remove the hybridized cDNA‐dye or cDNA‐antibody conjugates, and the labeling process is to tag the MIST array microbeads with specific fluorescent probes through DNA hybridization. The ordered combination of different colors composes codes for the identification of different ssDNA sequences and detected proteins on the MIST array elements.

Different from most conventional microarrays, the array elements in the MIST technique are randomly distributed but identifiable by successive cycles of quenching and labeling process. The whole procedure of multiplexed single‐cell measurement and analysis on MIST arrays is shown in **Figure** **1**. A highly compact monolayer of ssDNA encoded microbeads (ssDNA‐microbeads) is uniformly patterned on a glass cover slip (1 cm × 1 cm). Those ssDNAs are hybridized with complementary DNA (cDNA)‐antibody conjugates, so each microbead type is able to detect one species of protein via sandwich enzyme‐linked immunosorbent assay (ELISA). This monolayer cover slip is enclosed with polydimethylsiloxane (PDMS) microwells containing single cells to form a working microchip. During the on‐chip incubation, proteins secreted by single cells are captured by the anchored antibodies on the MIST array in each microwell. Fluorescence sandwich ELISA is used to detect the presence of various secreted proteins on thousands of arrays.

To know which protein is detected on a specific microbead, or the protein identity (ID), relies on the decoding process. Since the location of each microbead of any array is fixed, the fluorescent color on the same microbead can be recorded by a few successive processes of denaturation and hybridization of fluorophore tagged cDNAs (cDNA‐dye). Thus, after ELISA protein detection and imaging, the cDNA‐antibody conjugates are removed from the microbead surface using 1 m NaOH solution, leaving only ssDNAs on the surface for further hybridization. This successive hybridization (labeling) and denaturation (quenching) can be continuously executed for a few cycles. Eventually every microbead on the array exhibits ordered fluorescent colors, like an encrypted code, as predesigned on the combinatorial cDNA‐dye. Every code is corresponding to an ssDNA sequence as well as a type of antibody carried on the microbeads. This simple combinatorial labeling efficiently allows the number of detectable DNA oligo sequences (which correspond to protein species) to grow exponentially with the imaging round number. Theoretically, if M different colors are used for labeling in every staining cycle, and *N* cycles are performed, the multiplexity can reach as much as *M^N^*.

### 2.2 Characterization of MIST Array for Multiplex Barcoding

**Figure 2:**
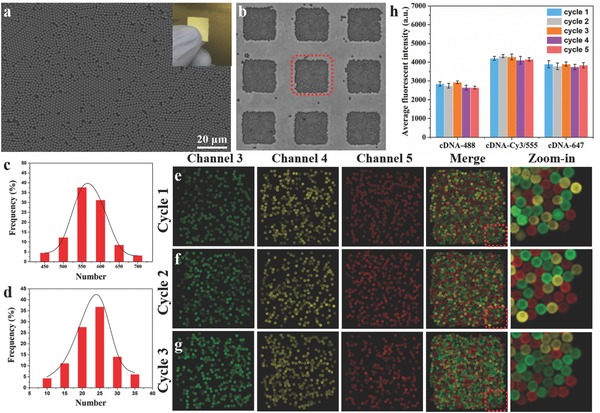
Large‐scale monolayer and MIST arrays formation and technique validation. a) Scanning electron microscopy image of patterned monolayer microbeads on a cover slip. Inset image shows a 1 cm × 1 cm MIST array on a cover slip. b) Formation of MIST arrays after mating the monolayer microbeads with PDMS microwells chip. c) Distribution of the numbers of microbeads on each MIST array. d) Distribution of the numbers of same kind microbeads on each array. e–g) Detection of 20 ssDNAs through decoding with three cycles and three colors of the marked array in inset (b). Both merged images and zoom‐in images are shown on the right. h) Fluorescence intensities after using the same fluorophore tagged cDNA in successive five cycles. The cDNA (Dʹ) were tagged with fluorophore Alexa 488, Cy3, and Alexa 647. 9 microarrays were imaged and quantitated for each condition. Error bars indicate standard deviation.

The large‐scale microbeads monolayer is patterned by an air/water interfacial self‐assembly method.^10^, ^11^, ^12^ As shown in **Figure** **2**a, the monolayer of ssDNA‐microbeads was hexagonally close‐packed on a coverslip. The glass substrate fully covered with microbead monolayer was cut to 1 cm × 1 cm for further experiment (Figure 2a, inset). This monolayer is segmented into more than ten thousand of microarrays after mating and detaching from a PDMS microwells stamp, with only a few microbeads left outside of microwell areas (Figure 2b). The clear shape of microarray is beneficial to overlapping of images in data processing. The number of microbeads across arrays follows Gaussian distribution (Figure 2c,d). Each MIST array at 50 µm × 50 µm patterned by 2 µm microbeads contains 450–700 microbeads which are capable of coding hundreds of analytes (Figure 2c). Each type of ssDNA‐microbeads is designed to be ≈25 copies per array (Figure 2d). This ensures that by random distribution each array always contains all types of ssDNA‐microbeads. We studied whether this copy number variation affects measurement error and protein quantification, where recombinant proteins at low and high amount in microwells were applied. Figure 5c shows the protein signals of microbeads by incubating with 0.1, 1, and 10 ng pL^−1^ IL‐8 standard protein across ≈500 microwells. The results show that the averaged fluorescent intensity is statistically uniform under variable number of microbeads in the microwells, which is consistent with the previous report.^13^

20 types of ssDNA‐microbeads were used to assess the MIST technology in multiplex detection (Figure 2e–g). The ssDNAs and their cDNAs were validated to have no crosstalk between any of noncomplementary pairs. A 3‐color × 3‐cycle method was employed to decode 20 targets on an array using cDNA‐dye probes (cDNA‐488, cDNA‐Cy5/555, and cDNA‐647, Figure S1, Supporting Information). We have predesigned a unique color code for each ssDNA‐microbead. In principle, 3‐color × 3‐cycle approach permits decoding of 27 different types of ssDNA‐microbeads. For each cycle, selected cDNA‐dye probes are mixed and applied to the arrays to fluorescently label every microbead through DNA hybridization. The fluorescent images of three different channels (green, yellow, and red) are taken, and subsequently all cDNA‐dye probes are dissociated by NaOH solution. The fluorescence in all channels is completely vanished, confirmed by imaging. Another cycle starts with the same procedure by different mixture of cDNA‐dye probes. All the microbeads in three cycles were stained by fluorophores (merge and zoom‐in images; Figure 2e–g). Figure 2h confirms the robustness of signal analysis on microbeads even after five cycles of hybridization and denaturation, as the fluorescence intensities are not statistically changed between cycles. Thus, much higher cycle number is practically achievable if an ultrahigh multiplexity is required.

### 2.3 Single‐Cell MIST Technology for Multiplexed Protein Detection

**Figure 3:**
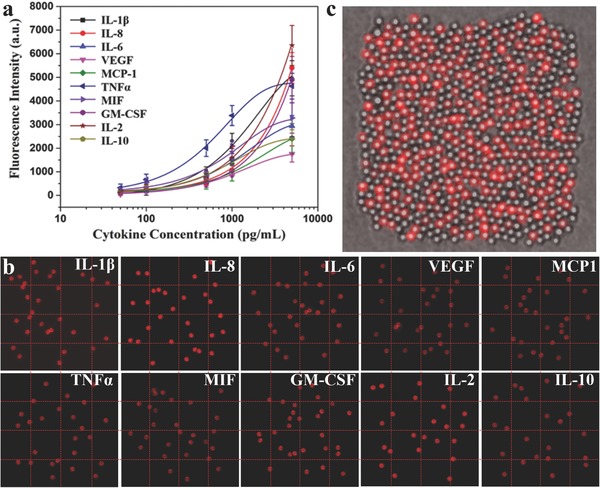
Sensitivity and crosstalk of the MIST array for multiplexed protein detection. a) Calibration curves for immunoassays performed on the MIST arrays using recombinant protein IL‐1β, IL‐8, IL‐6, VEGF, MCP1, TNFα, MIF, GM‐CSF, IL‐2, and IL‐10 at various concentrations. b) Crosstalk examination of detecting those ten proteins. One recombinant protein species was detected by sandwich ELISA at one time on the same MIST array. After quenching, the other microbeads on the same array selectively detect another protein. Grid was superimposed to facilitate visual comparison. c) Overlay of all fluorescence images from ten proteins detection merged with the bright field image.

Before single‐cell analysis, bulk test on the MIST arrays is demonstrated to detect ten cytokines using recombinant protein standards (IL‐1β, IL‐8, IL‐6, VEGF, MCP‐1, TNFα, MIF, GM‐CSF, IL‐2, and IL‐10). Those cytokines are involved in the critical macrophage functions including promotion and inhibition of inflammation, stimulation of leukocyte growth, and recruitment of other immune cells.^14^ The ssDNA‐microbead array was converted to an antibody array for protein detection through hybridization with cDNA‐antibody conjugates. By varying recombinant protein concentrations, the detection limits of the system is determined to be 43 pg mL^−1^ (IL‐1β), 55 pg mL^−1^ (IL‐8), 64 pg mL^−1^ (IL‐6), 103 pg mL^−1^ (VEGF), 72 pg mL (MCP‐1), 18 pg mL (TNFα), 65 pg mL^−1^ (MIF), 61 pg mL^−1^ (GM‐CSF), 12 pg mL^−1^ (IL‐2), 37 pg mL^−1^ (IL‐10), respectively (**Figure** **3**a), with a dynamic range of three to four orders of magnitude. Those detection limits and the dynamic ranges are fairly comparable to the data by conventional well‐plate method (provided by vendors). The variation of fluorescence intensities across multiple microbeads when measuring the same protein is determined to ≈7%, which is negligible compared to protein quantity change (Figure 5c). Crosstalk was examined by successively adding each type of protein standards and recording the microbead locations before quenching. As shown in Figure 3b,c, the locations of microbeads have no overlapping between any images.

The MIST technique is combined with PDMS microwells to analyze protein secretion by single cells. A model cell line THP‐1 is applied here to facilitate technology development and demonstrate its capability. THP‐1 monocytes can be differentiated into macrophages upon phorbol 12‐myristate 13‐acetate (PMA) stimulation, and further challenging with lipopolysaccharide (LPS) induces production of a variant of cytokines.^15^, ^16^ The LPS‐stimulated macrophage cell line is an ideal model system for single‐cell secretion analyses because they rapidly produce a large number of effector proteins, and the influence of cell cycle on heterogeneity is minimal due to postmitotic feature of differentiated macrophages.^6^, ^17^ The morphology change from suspension phenotype to adherent phenotype indicates the differentiation of monocytes to macrophages (Figures S2 and S3, Supporting Information).^18^

**Figure 4:**
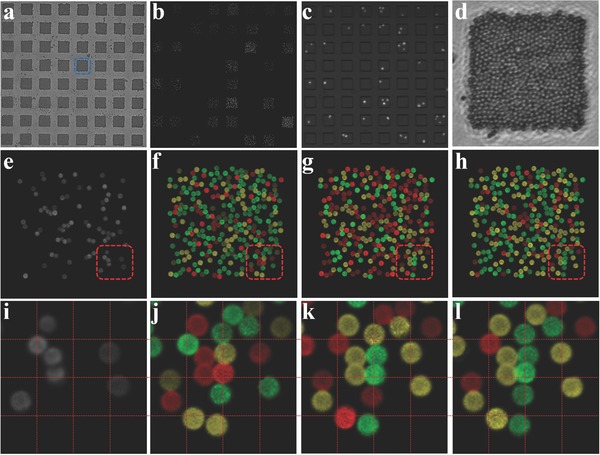
Single‐cell detection of ten proteins including IL‐1β, IL‐8, IL‐6, VEGF, MCP1, TNFα, MIF, GM‐CSF, IL‐2, and IL‐10 with MIST integrated microchip. a–e) Bright field, fluorescence images of a large‐scale MIST arrays after single‐cell detection and the corresponding merged image of microwells containing cells. Cells were labeled with Calcein AM fluorogenic dye. d) Bright field image and e) fluorescence image correspond to a representative MIST array (as marked in inset (a)) after detection. f–h) Overlapped fluorescence images of cycle f) 1, g) cycle 2, and h) cycle 3 for decoding the identity of each microbead. Each of the ten microbead groups carries a specific barcoded ssDNA sequence, or antibody type, for detection of one specific protein species. Excess microbead carrying non‐coding ssDNA was added and showed no fluorescence in all three channels. i–l) Zoom‐in images of the red rectangles in insets (e–h). Grids are superimposed to facilitate visual comparison.

For single‐cell assays, cells are loaded to the PDMS microwells before sealing them with antibody‐microbead arrays which are converted from ssDNA‐microbead arrays. The random seeding results in about 30% of 14 400 microwells filled with one cell when 10 000 cells are applied. The fluorescently labeled cells in microwells were imaged before and after incubation, and the location and cell number were recorded to match the final detection data (**Figure** **4**b,c). Clamping of the microchip prevents any leakage of cytokines between microwells during the 6 h incubation time (Figure S4, Supporting Information). A sufficient number of cytokines would be produced within 6 h according to our previous study and literature.^19^, ^20^ Longer incubation time may affect cell viability and stress cells to alter their functions, due to limited available nutrition in an enclosed microchamber. After incubation, the microbeads coverslip was separated from the PDMS microwells, and the arrays formed on the coverslip showed the projection of microwells. The protein secretion was detected by sandwich ELISA, and the same aforementioned decoding method was used to assign protein IDs to the detected signals.

Figure 4d–l shows an example of the decoding and ID assignment processes. About half of the ssDNA‐microbeads were converted to antibody‐microbeads before patterning. The bright field image (Figure 4d) is used as the reference for locating and mapping the microbeads in protein detection and in decoding. After single‐cell protein measurement, the microbeads in the fluorescence image (Figure 4e) are in different brightness because various number of cytokines is detected. The fluorescence within the periphery of every microbead is quantitated and averaged to represent its fluorescence intensity. Through decoding, every encoded microbead of the same array shows an ordered color change (Figure 4f–h,j–l; Figure S7, Supporting Information), giving the ID of the ssDNA or antibody on it. To the end, every encoded microbead has a fluorescence intensity and the corresponding ID. The collective information across all encoded microbeads of an array results in the secretion profile of a single cell.

### 2.4 Heterogeneity of Single THP‐1 Cells in Cytokine Production

**Figure 5:**
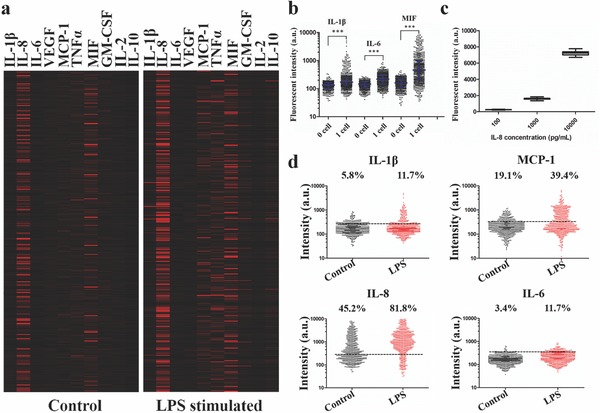
Single‐cell immune effector function profiling. a) Heatmaps of single‐cell protein profiles measured by the single‐cell MIST microchip on THP‐1 control cells and LPS stimulated cells. Each row represents a complete protein profile from a single‐cell measurement and each column is a protein of interest. b) Representative vertical scatter plots of IL‐1β, IL‐6, and MIF signal from one‐cell microwells compared to background levels from zero‐cell microwells. Every dot represents the average fluorescence intensity of all the microbeads of the same type in a microwell that contains either one cell or no cell. *P* values are 0.05 (*), 0.01 (**), and 0.001 (***), with 0.05 considered statistically significant. Blue bars are median values, and black bars are interquartile ranges. c) Fluorescent intensity of detecting IL‐8 standard proteins with different numbers of microbeads per microwell. Error bars represent the standard deviation of three repeats. d) Vertical scatter plots comparing single‐cell secretion of IL‐1β, IL‐8, MCP1, and IL‐6 at the control level (black dots) and upon LPS stimulation (red dots). The dash lines indicate calculated threshold from zero‐cell data.

We analyzed the cytokine secretion profiles of control and LPS stimulated THP‐1 cell populations. Bulk test reveals that the LPS stimulated macrophages significantly produce more cytokines than unstimulated cells (Figure S5, Supporting Information). Among all ten cytokines in the panel, six of them increase expression more than twofolds after stimulation. Two representative heatmaps in **Figure** **5**a permit visualization of vast heterogeneity and change of protein profiles from control macrophages to LPS stimulated macrophages. Each row of the heat map represents a single cell, and each column is a protein of interest. The single‐cell result in Figure 5a is consistent with the bulk. A significant percentage of cells produces MIF or IL‐8 even before stimulation. Expression of TNFα and MCP‐1 increases dramatically in the stimulated population, while IL‐2 and VEGF almost have no expression in either of the populations.

Similar to the isotype control of flow cytometry, we set a threshold for each protein by comparing the single‐cell data with the background. The zero‐cell datasets are used as an internal background reference where the mean intensity plus 2 × standard deviation of the signals in zero‐cell microwells is defined as the threshold (Figure 5b). This threshold permits quantification of the fraction of cells that positively secrete the proteins and excludes the data that are difficult to be distinguished from measurement noise. Eight out of ten proteins were considered detected, and six of them were significantly up‐regulated by LPS stimulation (Figure 5d; Figure S8, Supporting Information). Interestingly, for TNFα and IL‐6, LPS stimulation results in increase in both fraction of secreting cells and the secretion magnitude from those cells.^21^ However, some proteins including MIF and IL‐8 only increase the fraction, which indicates that a good portion of cells in the rest state became secretory after activation. Furthermore, we tested reproducibility of this technology by comparing two sets of single‐cell data from different microchips (Figure S9, Supporting Information). The distributions are found to be highly similar for three highly secreted proteins, and the percentages of positive secreting cells are varied only < 10%. This variation could be attributed to many factors, including the quality of antibody array, the proximity of cells to microbeads, data processing, batch‐to‐batch variation of cells and reagents.^17^, ^22^, ^23^, ^24^

### 2.5 Single‐Cell Analysis of Human Primary Monocyte‐Derived Macrophages

At last, we applied our MIST technique to investigate single‐cell cytokine functions of human primary monocyte‐derived macrophage cells. The monocytes from a healthy donor were induced to differentiate into an M1‐like phenotype (Figure S10, Supporting Information).^25^, ^26^, ^27^ Viability test found > 95% cells remain alive after 6 h on‐chip incubation. Using the same technique described above, we demonstrated that nine proteins were detected (Figure S11, Supporting Information). The overall secretion profile of human macrophages is fairly similar to THP‐1 cells, although the percentage of active cells is varied slightly (e.g., 50.7% IL‐8 secreting cells in primary macrophage vs 81.8% in THP‐1 cell line). To further exploit the rich single‐cell data, they were analyzed and visualized by t‐distributed Stochastic Neighbor Embedding (tSNE) algorithm.^28^, ^29^ This nonlinear algorithm allows for dimensionality reduction of high‐dimensional data into a space of two dimensions while still maintaining the data structure. The derived viSNE based on tSNE has been widely applied to visualize multiplexed mass cytometry data to discover subpopulations.

**Figure 6:**
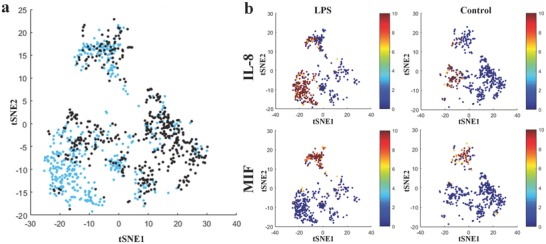
Functional heterogeneity and subpopulations of derived human macrophages. a) tSNE generated two‐dimensional projections of the single‐cell data of detecting ten secreted proteins. Each dot is a single macrophage cell that is not treated (black) or stimulated with LPS (blue). b) tSNE plots of individual proteins including MIF and IL‐8 under LPS stimulation condition and no treatment (control). (see tSNE for other proteins in Figures S11 and S12 in the Supporting Information).

The tSNE analysis reveals profound diversity of cells in cytokine production. All single cells (LPS stimulated and control) were mapped onto the same 2D tSNE plot (**Figure** **6**a). We are able to identify distinctive subpopulations that are dominated by IL‐8 secretion and MIF secretion. A quiescent subpopulation, which is the largest one in the control sample, can be also distinguished from other scattered subpopulations. Interestingly, the overall structure has not been shifted much after LPS stimulation. The MIF subpopulation is completely separated from others, and the heterogeneity is not changed significantly after stimulation.^6^ This data indicate that MIF secretion is conservative function to macrophages. On the contrary, a significant conversion of quiescent cells to IL‐8 secreting cells is observed on the tSNE plot (Figure 6b). This subpopulation contains polyfunctional cells as it also produces TNFα and IL‐10 (Figure S12, Supporting Information). Except for MIF and IL‐8, other proteins are scattered in various clusters (Figure S13, Supporting Information) with and without LPS stimulation, indicating the high heterogeneity of primary macrophage cells.

## 3 Discussion

The highly multiplexed and quantitative molecular profiling of single cells and clinical tissue specimens are only emerging in recent years. For the single‐cell cytokine secretion measurement, essentially only the barcode chips desirably cover the wide spectrum of functionally diverse cytokines. Evolved from barcode chips, the MIST technique we introduce here gives rise to high multiplexity by a simpler approach and is compatible with available equipment that most biological laboratories already have. More importantly, it is highly compact due to small size of microbeads, potentiating the ultrahigh multiplexed assays of single‐cell proteins. For example, by simply designing more barcode ssDNA probes and cDNA‐antibody conjugates, the same architecture of the current technology can measure up to 40 different cytokines. Noticeably, the other barcode chips in our group and those in Fan's group have microchamber volumes at least eight times larger for single‐cell cytokine assays.^6^ Therefore, without losing much sensitivity, this strategy by expansion of microarray size and microwell volume can further increase multiplexity to hundreds. The complexity and experimental time are not increased proportionally, as two more successive decoding than what was performed in this report can reach 243 (3^5^) multiplexity, with only 3 h more experiment. The other strategy to further improve multiplexity is to use smaller size of microbeads, as long as the individual microbeads are > 0.4 µm and are easily distinguishable by fluorescence microscope. The even smaller microbeads such as 0.2 µm as we tested pose challenges of decoding if they are adjacent to each other. Noteworthily, our successive decoding is naturally error‐free, in contrast to multiplex RNA fluorescence in situ hybridization techniques.^30^, ^31^ That is because each microbead has numerous number of probes that are sufficient to ward off mistargeting. The quality of array is influenced by the patterning approach and ssDNA loading, although the air/water interfacial self‐assembly method is very efficient to generate a super‐large scale of microbead monolayer. Though the data from those replicates can be removed by computational programs, a more uniform patterning technique would facilitate the development of this technology towards industrialization and commercialization.

With the single‐cell MIST technology, the vast heterogeneity of cytokine production has been observed. The single‐cell cytokine analysis can thus provide deep insight of biological diversity of immune cells which play the central role in inflammation. The phenotypically similar cells still possess very distinguishable functions, prompting more questions about the cellular immunity. The further investigation may better involve analysis of intracellular proteins and surface markers. Although such a study is beyond the scope of this report, the integration of the MIST technique with a modified single‐cell microchip for intracellular protein assays is highly feasible, by replacing our flow‐patterned barcode array with the MIST array.

In summary, we have developed a unique and technically simple approach for highly multiplexed single‐cell protein profiling. This MIST technique is comprised of interconvertible DNA‐antibody arrays and successive decoding process, ideally overcoming the fundamental limitations of the prevailing microfluidic‐based methods. The integration of the MIST technique with a single‐cell microwells chip permits high‐throughput, high‐multiplex detection of cytokines secreted by LPS challenged macrophages. Application of the technology results in the distinguishment of functionally heterogeneous subpopulations of macrophage cell line and human primary macrophages. We envision that further development of MIST technology, especially with automated labeling‐imaging instrument, would make it have tremendous potential for biomedical applications, including immune response monitoring in both preclinical and clinical applications.

## 4 Experimental Section

*Conjugation of ssDNA‐Microbeads*: The amine‐microbeads were modified with trans‐cyclooctene (TCO), and the amine ended ssDNAs were separately functionalized with methyltetrazine (Tz). Through Tz‐TCO click reaction, the ssDNAs were covalently coated on microbeads. Specifically, 2000 µL of 2% amine bearing microbeads (Thermo Fisher Scientific) were washed with Milli‐Q (MQ) water and then mixed with 20 µL of 0.2 mg µL^−1^ TCO‐PEG_12_‐NHS (Click Chemistry Tools LLC.) solution for 4 h. The functionalized microbeads were purified by centrifugation three times to remove unbound TCO, and were resuspended to the original concentration. Each of 50 µL amine ended oligo ssDNAs (denoted as D, E, F, G, H, I, J, K, L, M, N, O, P, Q, S, U, Z, BB, CC, HH; Integrated DNA Technologies, IDT) at 1 × 10^−3^ m was mixed with 5 µL of 0.2 mg µL^−1^ sulfo‐Tz‐NHS (Click Chemistry Tools LLC.) solution for 4 h. Those ssDNAs were validated without crosstalk on conventional printed microarrays. The Tz‐DNA conjugates were purified with Zeba desalting columns (Thermo Fisher Scientific). Finally, 100 µL of prepared TCO modified microbeads were mixed with 50 µL of Tz modified ssDNA conjugates for 4 h, followed by purification and collection using centrifugation. The obtained ssDNA‐microbeads were stored in 4 °C for further use.

*Preparation of cDNA‐Antibody Conjugates*: The conjugation of cDNA with antibody was described before.^19^ In brief, the 200 × 10^−6^ m complementary oligonucleotides were modified with 4‐formylbenzamide group (SFB; Solulink), while the 3.3 × 10^−6^ m antibodies were modified with 6‐hydrazinonicotinamide (s‐HyNic; Solulink). The formation of the Schiff base by reaction of SFB and s‐HyNic at pH 6.0 coupled the antibody to oligonucleotides. The conjugates were purified using high‐performance liquid chromatography workstation and stored in 4 °C for further use.

Antibodies used in conjugation for single‐cell assays were listed in Table S1 in the Supporting Information. Ten capture antibodies targeting IL‐1β, IL‐8, IL‐6, VEGF, MCP‐1, TNFα, MIF, GM‐CSF, IL‐2, and IL‐10 were conjugated with the cDNA Dʹ, Eʹ, Fʹ, Gʹ, Hʹ, Iʹ, Jʹ, Mʹ, Nʹ, and Oʹ (IDT), respectively. The DNA sequences used in this study are listed in Table S2 in the Supporting Information.

*Preparation of cDNA‐Dye Conjugates*: Three fluorescent colors of cDNA‐dye conjugates were prepared for decoding of 20 different ssDNA‐microbeads. The selected cDNAs were directly reacted with dye‐NHS ester, and some cDNA‐dye were purchased from IDT. For green color fluorescence tagging, 200 µg of DyLight 488 ester (Thermo Fisher Scientific) was dissolved in 5 µL dimethylformamide (DMF) and was then added to 10 µL of 40 × 10^−6^ m cDNA for overnight reaction. For yellow color fluorescence tagging, 50 µg of Alexa‐555 ester (Thermo Fisher Scientific) was dissolved in 10 µL phosphate buffered saline (PBS) solution, and was mixed with 10 µL of 40 × 10^−6^ m cDNA for overnight reaction. For red color fluorescence tagging, 50 µg of Alexa‐647 ester was dissolved in 10 µL PBS solution, followed with the same incubation with cDNA overnight. All conjugates were purified by 7K MWCO Zeba column and diluted to 10 × 10^−6^ m for further use.

*Fabrication of the Microwell Chip*: The PDMS microwells were fabricated using standard soft‐lithographic technique.^32^ The features on a chrome photomask printed by Front Range Photomask were patterned on a 4″ silicon wafer using photoresist SU‐8 2025 (Microchem). PDMS prepolymer (Sylgard 184; Dow Corning) was mixed in a ratio of 10:1, and subsequently casted on this lithographically patterned mold. After curing at 75 °C for 1 h, the PDMS replica containing 14 400 microwells (length: 50 µm; width: 50 µm; height: 50 µm) was separated from the mold and kept in a vacuum chamber for overnight.

*Patterning Monolayer of ssDNA‐Microbeads*: The ssDNA‐microbeads monolayer was prepared on a cleaned glass coverslip (1 cm × 1 cm) by an air/water interfacial self‐assembly strategy.^10^, ^11^, ^12^, ^33^ Briefly, 2% ssDNA‐microbeads suspension in water were mixed with ethanol at a volume ratio of 1:1. The glass coverslip was covered with 100 µL of MQ water to form a thin layer of water phase, before slowly dropping 20 µL ssDNA‐microbeads mixture in ethanol solution onto the water surface. A microbead monolayer was formed spontaneously by self‐assembly at the air‐water interface. About 5 µL of 2% sodium dodecyl sulfate (SDS; Thermo Fisher Scientific) was introduced dropwise to consolidate the particles to form closely packed colloidal crystal. After water evaporation, the monolayer was settled down on the glass coverslip. Baking at 75 °C for 15 min effectively enhanced the attachment of microbeads to the coverslip.

*Decoding, Calibration, and Validation*: Lifting of excessive microbeads by PDMS microwell stamp gives rise to thousands of 50 µm × 50 µm barcode arrays. The arrays were transferred to a transparent packing tape that was fixed on a glass coverslip to prevent any loss of microbeads during the process. After blocking nonspecific binding with 1% bovine serum albumin (BSA) in PBS for 1 h, 100 µL of whole panel cDNA‐dye conjugates (Cocktail I; Table S3, Supporting Information) at 0.5 × 10^−6^ m was applied on the arrays and incubated for 1 h. The arrays were washed by 1% BSA in PBS and subsequently imaged under three fluorescence channels and bright field. Incubation with 1 m NaOH for 5 min was used to dissociate double strands and remove the cDNA‐dye conjugates. In the second cycle and the third cycle, cDNA‐dye conjugates panel cocktail II and cocktail III were used to selectively label microbeads, following the same procedure of the first cycle. The same arrays were imaged throughout all cycles and were overlapped to determine the order of fluorescent color change for each microbead.

The ssDNA arrays were converted to antibody arrays by incubating the cDNA‐antibody conjugates at 2.5 µg mL^−1^ on the coverslip at 37 °C. Conversion was always done immediately preceding on‐chip protein detection assays. For calibration of the detection system, only one recombinant protein in a series of concentrations from 50 pg mL^−1^ to 5 ng mL^−1^ was added at a time. The fluorescence intensity of array elements was recorded and digitalized. To examine crosstalk and orthogonality of detection, repetitive sandwich ELISA experiments were performed on the same array for ten times, each with one recombinant protein at 10 µg mL^−1^. After every ELISA experiment, all the cDNA‐antibodies and the fluorescence conjugates were removed on the array by NaOH solution. The same cocktail of ten cDNA‐antibodies was applied to the same array for the next detection of recombinant protein.

*Cell Culture, Differentiation, and LPS Stimulation*: The human monocyte cell line THP‐1 was gifted by Dr. Jian Zhu at University of Rochester. Cells were routinely cultured in RPMI‐1640 medium in T75 cm^2^ flask supplemented with 10% (v/v) fetal bovine serum (FBS) and 50 × 10^−6^ m 2‐mercaptoethanol at 37 °C in 5% CO_2_ incubator. For differentiation into macrophage cells, the monocytes were incubated with 50 ng mL^−1^ PMA (Sigma‐Aldrich) for 48 h in complete medium, followed by culturing in the complete medium without PMA for 24 h. Cells were trypsin treated to be in suspension and then stained with 1 × 10^−6^ m Calcein AM (Thermo Fisher Scientific) for single‐cell experiments. LPS at 1 µg mL^−1^ was supplied in the complete medium for all stimulation experiments, while control cells were those without LPS treatment. All cultures were incubated at 37 °C under 5% CO_2_ atmosphere.

Human primary CD14+ monocyte was purchased from PromoCell, and was further differentiated into macrophages by culturing with RPMI‐1640 complete medium (supplemented with 20% (v/v) FBS, 100 U mL^−1^ of penicillin, and 100 µg mL^−1^ streptomycin) containing with M‐CSF (50 ng mL^−1^; R&D) for 72 h and then RPMI‐1640 complete medium for 96 h. The macrophages were then collected with trypsin treatment for single‐cell experiment. With (LPS) and without (control) 1 µg mL^−1^ LPS were performed in our experiment. For viability validation, cells without fluorescence staining were loaded to microwells before incubation. They were labeled with Calcein AM after incubation and splitting the PDMS microwells from the array. The fluorescent cells were regarded as live cells.

*Multiplexed Single‐Cell Protein Detection*: The encoded ssDNA array was converted to antibody array first by incubation with a cocktail of cDNA‐antibody conjugates for 1 h. The microwell side of PDMS replica was meanwhile treated with plasma (Harrick Plasma) for 1 min and incubated with 50 µL of 50 µg mL^−1^ collagen (Corning) for 30 min. After cleaning the PDMS by PBS, ≈10 000 cells in RPMI complete medium were dropped on the microwells and incubated at 37 °C for 20 min. The excess cell suspension outside of microwells was removed by gently scraping. A coverslip carrying antibody array was carefully mated with the PDMS microwell stamp, and the assembly was placed in a clamp to completely isolate cells in the microwells. Cells were incubated in a 37 °C incubator for 6 h, and their locations in microwells were recorded. The array coverslip was separated from the PDMS microwell stamp and was washed with 1% BSA/PBS before incubation with 200 µL of biotinylated detection antibodies (20 µL for each kind of ten antibodies) for 2 h. After washing with 1% BSA in PBS, 100 µL of streptavidin‐alexa 647 (Thermo Fisher Scientific) at 10 µg mL^−1^ was added onto the array and incubated for another 1 h. Finally, the coverslip was washed with 1% BSA in PBS and 0.05% Tween 20 in PBS for three times.

The array was imaged using fluorescence microscopy (Olympus IX73) and was air‐dried, immediately followed by transferring to a transparent packing tape fixed on a glass coverslip. 50 µL of 1 m NaOH solution was applied to remove fluorescence and denature cDNA fragment for 5 min, followed by thoroughly wash by 1% BSA/PBS. The same decoding method described above was employed to relate detected protein signaling with protein ID using ten different cDNA‐dye conjugates (cycle 1: Dʹ‐488, Eʹ‐488, Fʹ‐Cy3, Gʹ‐Cy3, Hʹ‐647, Iʹ‐647, Jʹ‐488, Mʹ‐488, Nʹ‐488, Oʹ‐555; cycle 2: Dʹ‐647, Eʹ‐647, Fʹ‐647, Gʹ‐488, Hʹ‐488, Iʹ‐Cy3, Jʹ‐Cy3, Mʹ‐Cy3, Nʹ‐647, Oʹ‐647; and cycle 3: Dʹ‐Cy3, Eʹ‐647, Fʹ‐647, Gʹ‐488, Hʹ‐488, Iʹ‐Cy3, Jʹ‐Cy3, Mʹ‐488, Nʹ‐488, Oʹ‐555)

*Fluorescence Microscopy Imaging*: An Olympus IX73 inverted fluorescence microscopy was used to take images of barcode arrays and cells in microwells or well plates. The green fluorescence, yellow fluorescence, and red fluorescence were taken with filter sets: a green fluorescein filter set (U‐FF, Olympus; excitation filter 450–490 nm, dichroic 500 nm long pass, emission 520 nm long pass), a yellow Cy3 filter set (U‐FF, Olympus; excitation filter 528–553 nm dichroic 565 nm long pass, emission 590–650 nm), and a red Cy5 filter set (U‐FF, Olympus; excitation filter 590–650 nm, dichroic 660 nm long pass, emission 665–740 nm). Microscope objectives (UPlanApo, Olympus, 4X/0.16; UPlanFLN, Olympus, 10X/0.30/Ph1; UCPlanFLN, Olympus, 20X/0.70/Ph2) were used to collect fluorescence light. A digital camera (Zyla sCMOS, Andor) mounted on the microscope was used to capture images using Andor SOLIS software.

*Data Analysis and Statistics*: The fluorescence images were digitalized, and the locations of microbeads were automatically identified by laboratory developed MATLAB programs. The programs also integrated the fluorescence intensity of every microbead for protein detection. Photoshop was used to overlap multiple decoding images to extract the order of fluorescent color change for each microbead. Matching the color order with the proteins in the decoding map determined the ID of the microbeads. Then the microbeads detecting the same protein were grouped and their fluorescence intensities were averaged for each array. The same procedure was repeated for each of ≈500 arrays which also carried the information of cell number per microwell or array. To the end, a dataset in table was generated including cell number (zero or one cell) and the corresponding protein detection signal.

The protein data in zero‐cell microwells/arrays provided a measure of antibody‐specific background, similar to isotype control in flow cytometry. The threshold was set as mean intensity plus 2 × standard deviation of the signals in zero‐cell microwells. Protein signal of any cell above the threshold was regarded as true secretion of this particular protein. Prism (GraphPad) was used to statistically compare zero‐cell data versus one‐cell data and generated plots. Unpaired, two‐tailed *t*‐test was used to determine statistically significant differences. A *P* value less than 0.05 is considered statistically significant and is denoted with *, while ** and *** represent *P* < 0.01 and *P* < 0.001, respectively. The t‐distributed Stochastic Neighbor Embedding (tSNE) algorithm is a built‐in function of Matlab 2017 (MathWorks). Colormap indicates the protein expression level.

## Conflict of Interest

The authors declare no conflict of interest.

## Supporting information

Supplementary
