# Supplementary material for: Highly Multiplexed Single‐Cell Protein Profiling with Large‐Scale Convertible DNA‐Antibody Barcoded Arrays
Source: Adv Sci (Weinh). 2018 Aug 2;5(9):1800672. doi: 10.1002/advs.201800672 (PMC6145231; doi:10.1002/advs.201800672)
Supplement: Supplementary file 1 — Supplementary [file ADVS-5-1800672-s001.pdf]

Copyright WILEY-VCH Verlag GmbH & Co. KGaA, 69469 Weinheim, Germany, 2018.

ADVANCED SCIENCE
Open Access

Supporting Information

for *Adv. Sci.,* DOI: 10.1002/advs.201800672

Highly Multiplexed Single-Cell Protein Profiling with Large-Scale Convertible DNA-Antibody Barcoded Arrays

*Peng Zhao, Sirsendu Bhowmick, Jianchao Yu, and Jun Wang**

Supplementary Information

Highly Multiplexed Single-Cell Protein Profiling with Large-Scale Convertible DNA-Antibody Barcoded Arrays

*Peng Zhao, Sirsendu Bhowmick, Jianchao Yu, and Jun Wang**

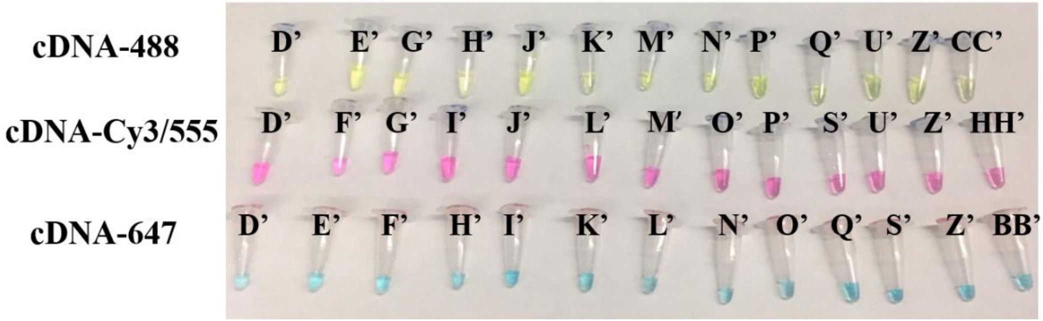

Figure S1. List of cDNA-dye conjugates. The upper, middle and bottom rows are the conjugates of cDNA with Dylight 488 (labeled as cDNA-488), cDNA-Cy3 and cDNA-Alexa Fluor 555 (cDNA-555), and cDNA with Alexa Fluor 647 (cDNA-647), respectively.

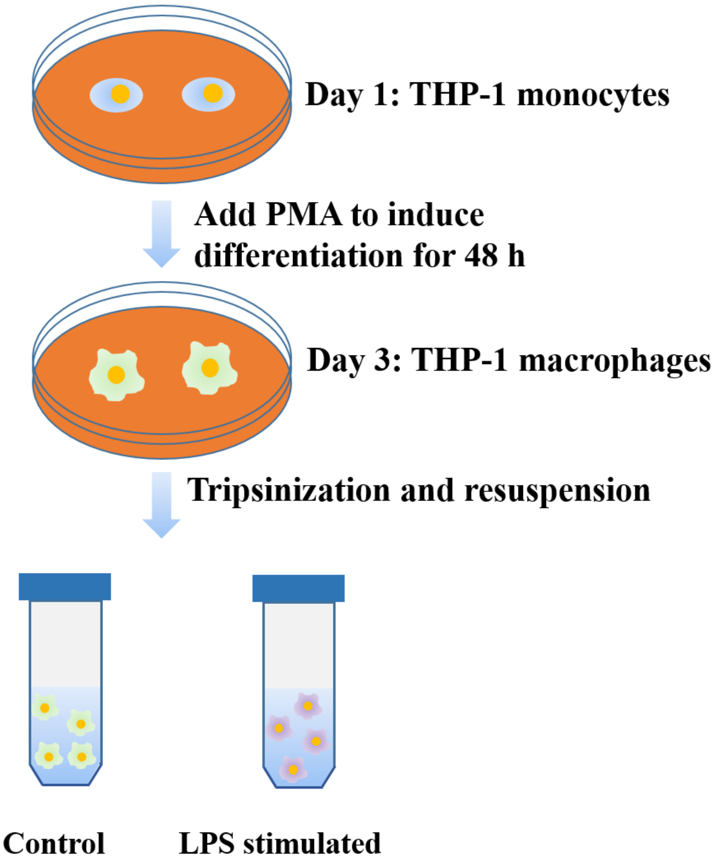

Figure S2. Procedure of preparing THP-1 derived macrophages for single-cell experiment. THP-1 monocytes were maintained in a Petri dish. After 3 days of differentiation by adding PMA, the macrophages were collected with trypsin. Half of the cells were directly measured by the single-cell chip as control, and the other one was stimulated by LPS before single-cell assay.

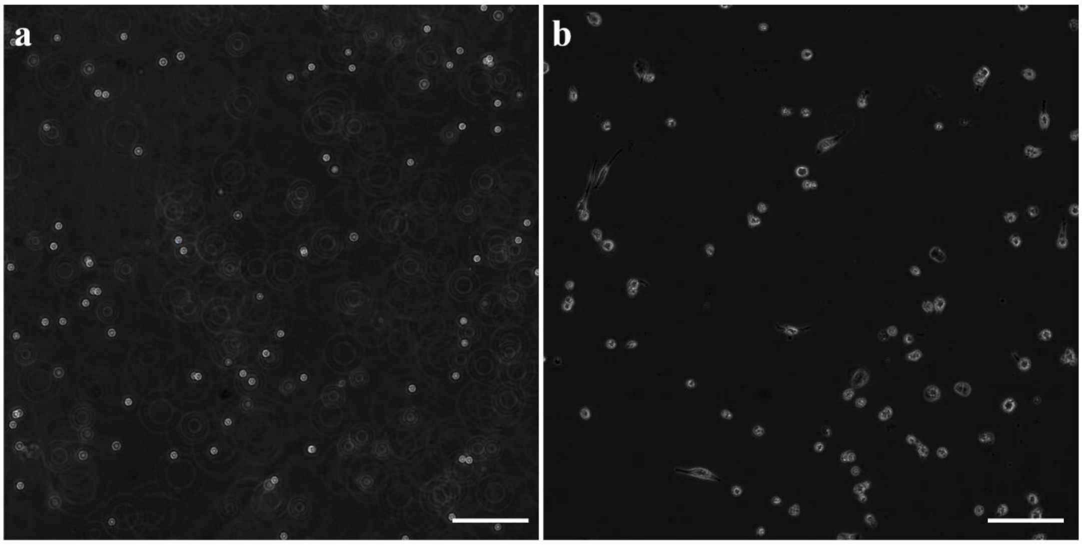

Figure 3. Morphology changes of THP-1 monocytes upon PMA induction. (a) Monocytic THP-1 cells before induction. (b) Macrophage-like THP-1 cells after PMA treatment for 48 h. The morphology change from suspension to adherent phenotypes was exhibited during the process. Scale bar: 100 μm.

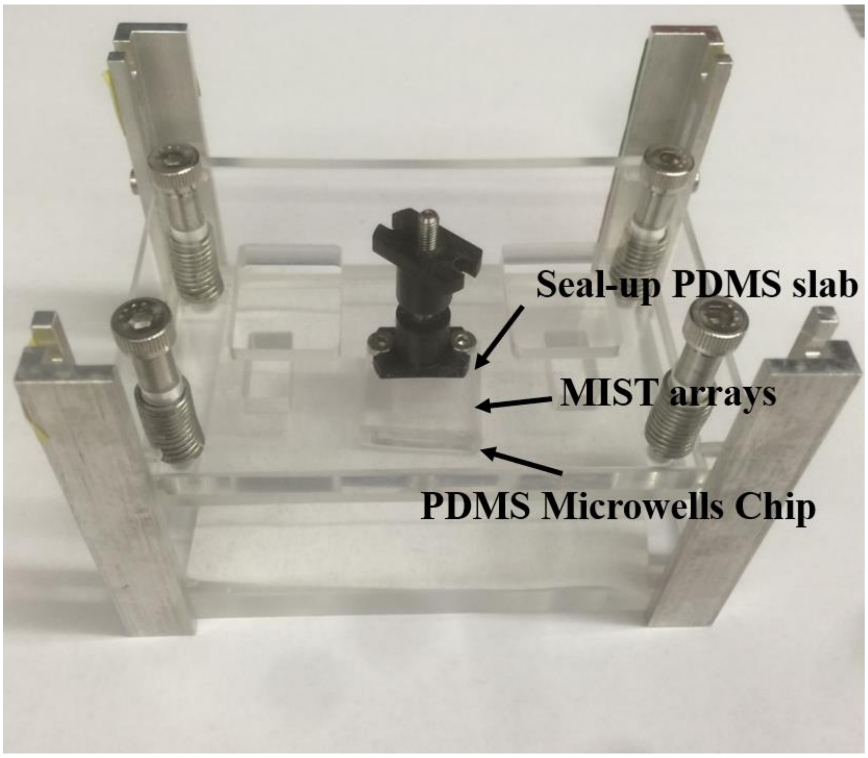

Figure S4. Photo of a complete single-cell assay setup. A clamp with adjustable screws is used to pressurize the PDMS microwells and the MIST array assembly to seal the microwells.

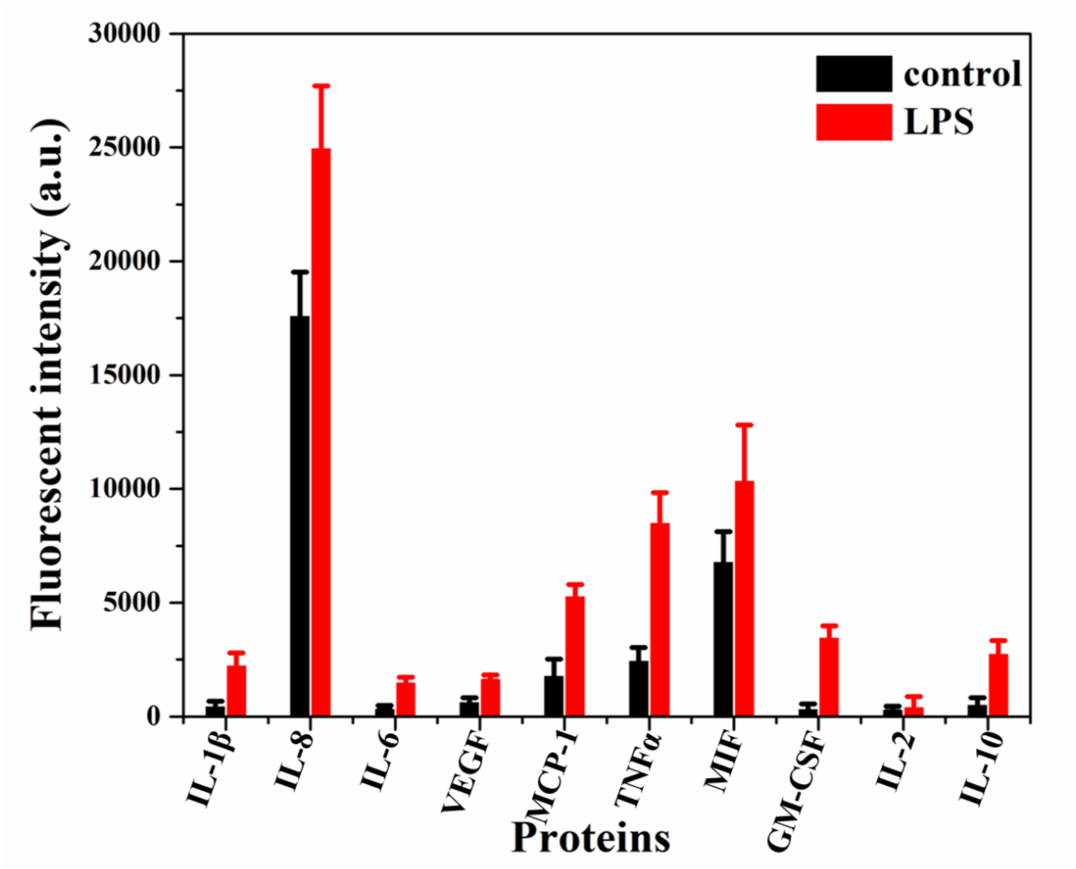

Figure S5. Protein secretion level of differentiated THP-1 macrophages. Both control and LPS stimulated macrophages at 10,000 cells/ml were cultured in 96-well plates for 6 h, and the supernatants were collected for conventional ELISA assays to quantitate protein concentrations.

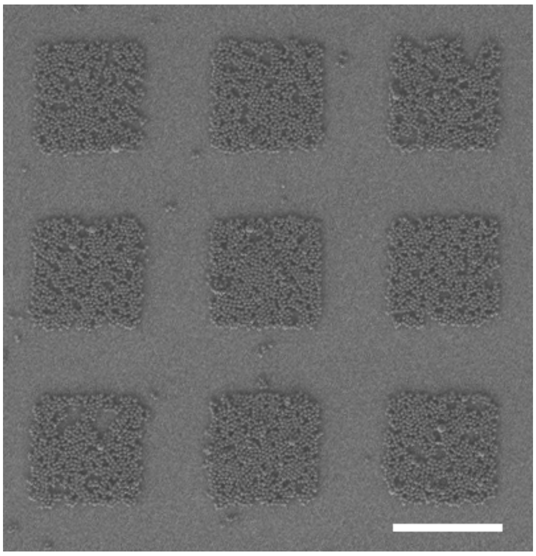

Figure S6. SEM image of MIST arrays. Scale bar: 50 μm.

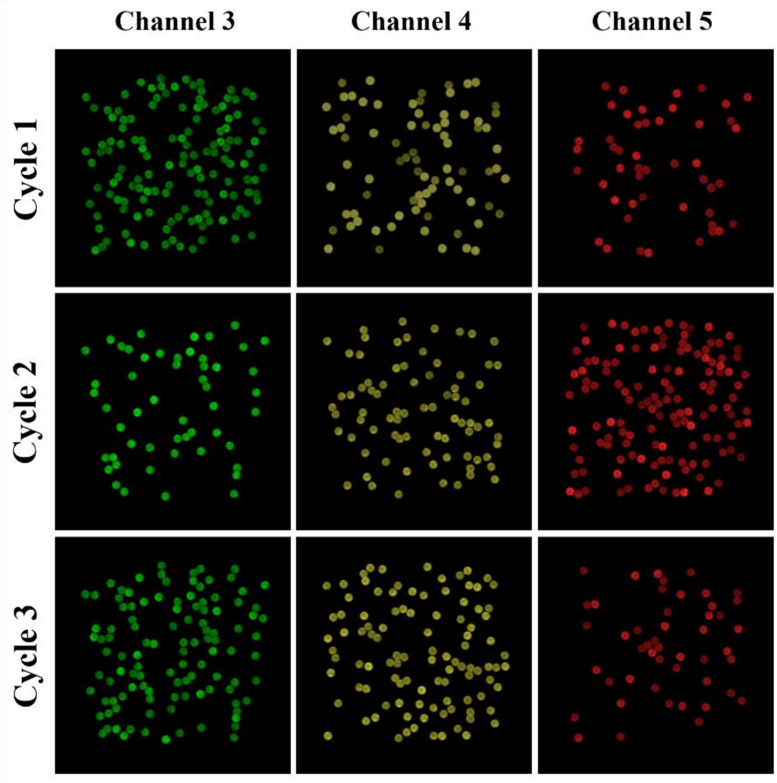

Figure S7. Fluorescent images corresponding to overlapped Figure 4f (cycle 1), g (cycle 2) and h (cycle 3).

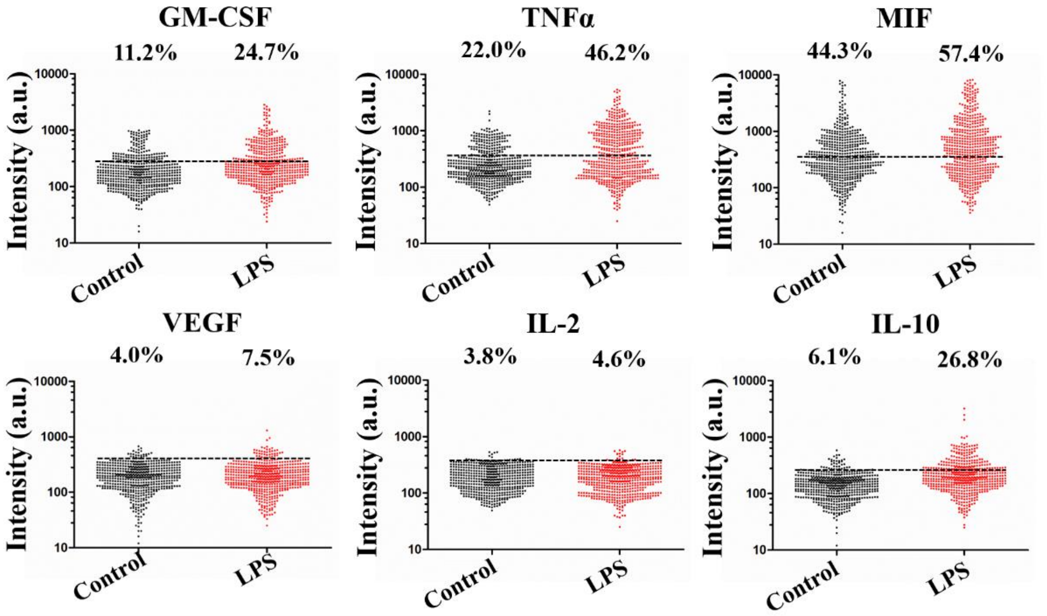

Figure S8. Vertical scatter plots for protein secretion levels and frequency in single cells for the proteins other than those in Figure 5d. The figures compare protein secretion levels with (red dots) and without (black dots) LPS stimulation. The dashed line marked as threshold (zero-cell average data + 2 SD).

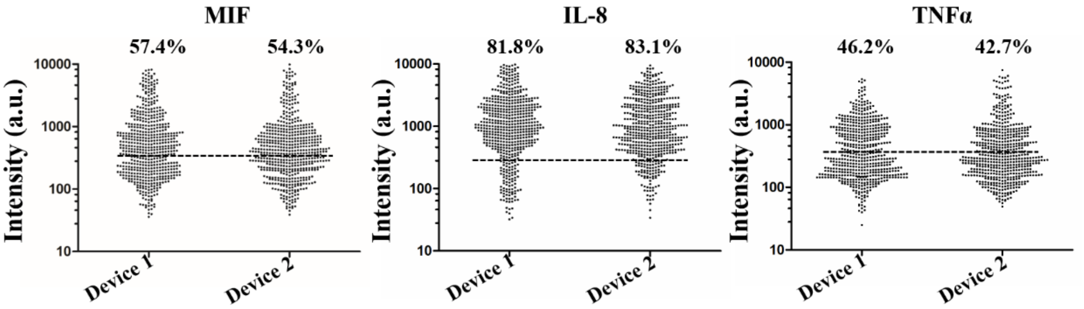

Figure S9. Dot plots comparison for three proteins data sets from two experiments performed in parallel. All the conditions for the whole experimental process were the same.

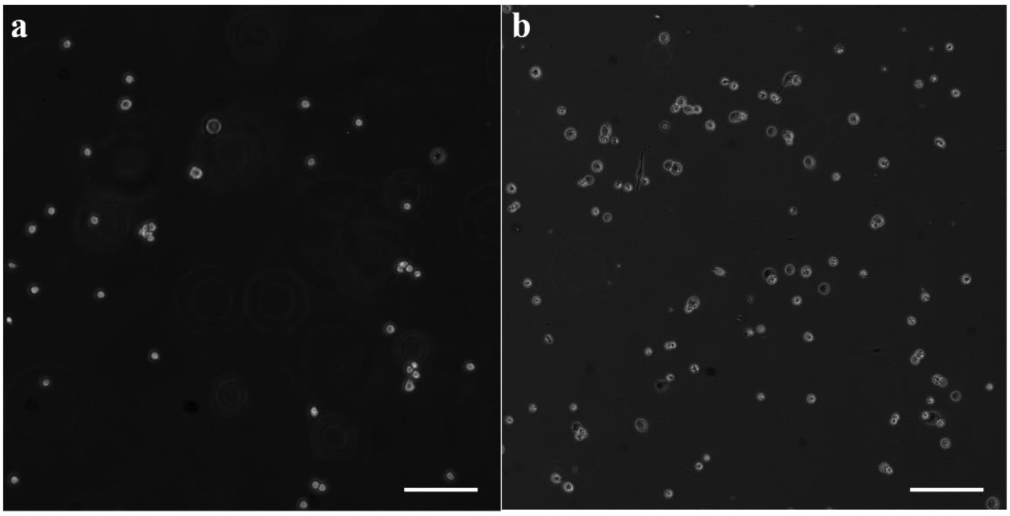

Figure S10. Morphology changes of human primary monocytes upon M-CSF induction. (a) Human primary monocytes before induction. (b) Macrophage-like primary cells after M-CSF treatment for 7 days. The morphology change from suspension to adherent phenotypes was clearly observed during the process. Scale bar: 100 μm.

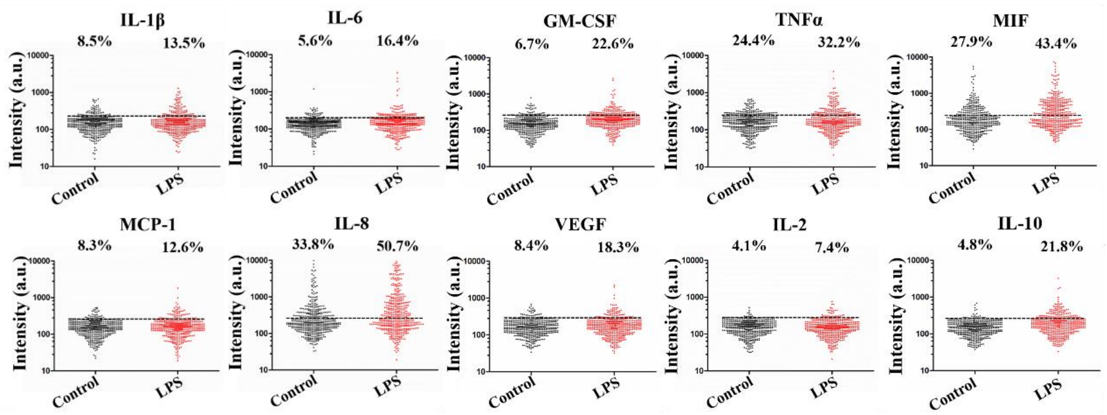

Figure S11. Vertical scatter plots for protein secretion levels and frequency in single human primary cells. The figures compare human primary cells protein secretion levels with (red dots) and without (black dots) LPS stimulation. The dashed line marked as threshold (zero-cell average data + 2 SD).

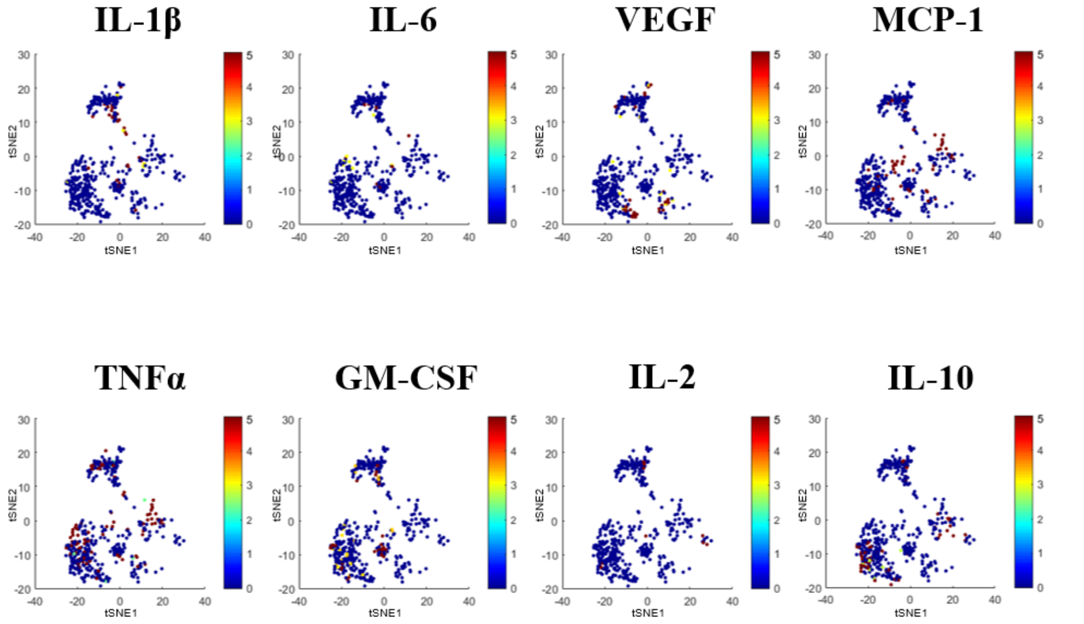

Figure S12. tSNE plots of individual proteins including IL-1β, IL-6, VEGF, MCP-1, TNFα, GM-CSF, IL-2 and IL-10 under LPS stimulation condition. The other two proteins (IL-8 and MIF) are analyzed in Figure 6b. The colormaps are linear with protein expression levels.

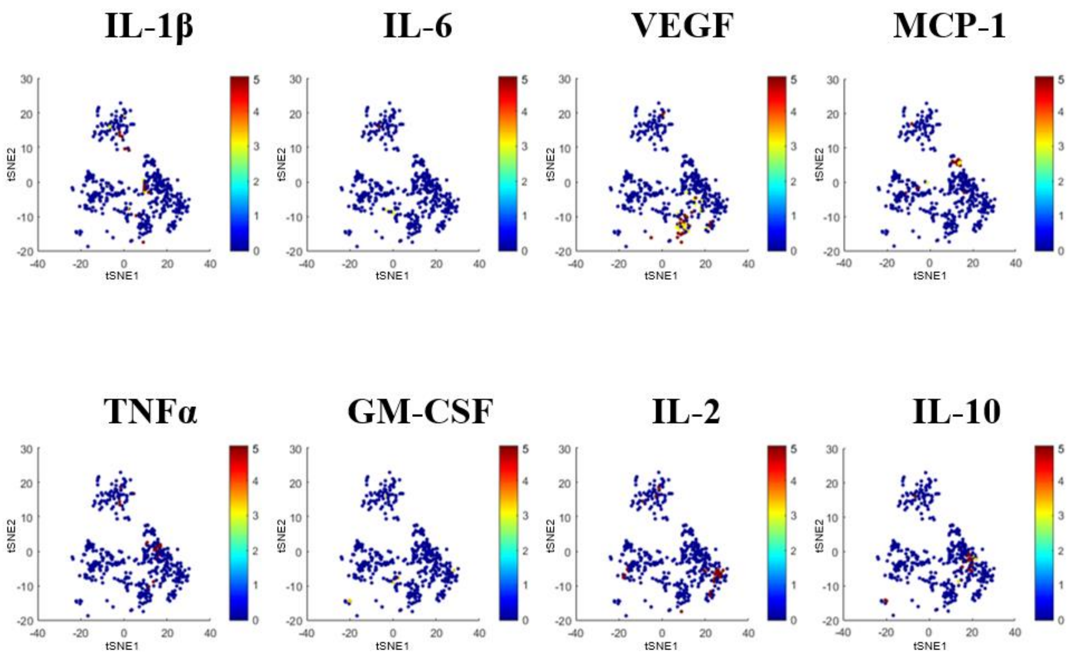

Figure S13. tSNE plots of individual proteins including IL-1β, IL-6, VEGF, MCP-1, TNFα, GM-CSF, IL-2 and IL-10 without stimulation. The other two proteins (IL-8 and MIF) are analyzed in Figure 6b. The colormaps are linear with protein expression levels.

Table S1. List of antibodies in this study and their vendor information.

Protein	Capture Antibody (Catalog/Company)	Detection Antibody (Cataloy/Company)
IL-1β	14-7018-81/eBioscience	13-7016-81/eBioscience
IL-8	431501/Biolegend	431501/Biolegend
IL-6	D6050/R&D	D6050/R&D
VEGF	AHG0114D/Invitrogen	AHG9119D/Invitrogen
MCP-1	505901/Biolegend	502608/Biolegend
TNFα	502802/Biolegend	502903/Biolegend
MIF	525502/Biolegend	525503/Biolegend
GM-CSF	DY215/R&D	DY215/R&D
IL-2	DY202/R&D	DY202/R&D
IL-10	DY217/R&D	DY217/R&D

Table S2. List of all the oligonucleotides for conjugation.

Oligo Name	Oligo Sequence
D	5'-/5AmMC6/AAA AAA AAA AAA AAT GGT CGA GAT GTC AGA GTA-3'
E	5'-/5AmMC6/AAA AAA AAA AAA AAT GTG AAG TGG CAG TAT CTA-3'
F	5'-/5AmMC6/AAA AAA AAA AAA AAT CAG GTA AGG TTC ACG GTA-3'
G	5'-/5AmMC6/AAA AAA AAA AAA AGA GTA GCC TTC CCG AGC ATT-3'
H	5'-/5AmMC6/AAA AAA AAA AAA AAT TGA CCA AAC TGC GGT GCG-3'
I	5'-/5AmMC6/AAA AAA AAA AAA ATG CCC TAT TGT TGC GTC GCA-3'
J	5'-/5AmMC6/AAA AAA AAA AAA ATC TTC TAG TTG TCG AGC AGG-3'
K	5'-/5AmMC6/AAA AAA AAA AAA ATA ATC TAA TTC TGG TCG CGG-3'
L	5'-/5AmMC6/AAA AAA AAA AAA AGT GAT TAA GTC TGC TTC GGC-3'
M	5'-/5AmMC6/AAA AAA AAA AAA AGT CGA GGA TTC TGA ACC TGT-3'
N	5'-/5AmMC6/AAA AAA AAA AAA AGT CCT CGC TTC GTC TAT GAG-3'
O	5'-/5AmMC6/AAA AAA AAA AAA ACT TCG TGG CTA GTC TGT GAC-3'
P	5'-/5AmMC6/AAA AAA AAA AAA ATC GCC GTT GGT CTG TAT GCA-3'
Q	5'-/5AmMC6/AAA AAA AAA AAA ATA AGC CAG TGT GTC GTG TCT-3'
S	5'-/5AmMC6/AAA AAA AAA AAA AGC GTG TGT GGA CTC TCT CTA-3'
U	5'-/5AmMC6/AAA AAA AAA AAA ATA TGG GTC TTG CTG ATA CGC-3'
Z	5'-/5AmMC6/AAA AAA AAA AAA ACT CTG TGA ACT GTC ATC GGT-3'
BB	5'-/5AmMC6/AAA AAA AAA AAA AAG TCT GAT CCC ATC GCG TAT-3'
CC	5'-/5AmMC6/AAA AAA AAA AAA AGA GGT CAG TTC ACG AAG CTC-3'

HH	5'-/5AmMC6/AAA AAA AAA AAA AGC ACT AAC TGG TCT GGG TCA-3'
D'	5'-/5AmMC6/AAA AAA AAA AAA ATA CTC TGA CAT CTC GAC CTC-3'
E'	5'-/5AmMC6/AAA AAA AAA AAA ATA GAT ACT GCC ACT TCA CAT-3'
F'	5'-/5AmMC6/AAA AAA AAA AAA ATA CCG TGA ACC TTA CCT GAT-3'
G'	5'-/5AmMC6/AAA AAA AAA AAA AAA TGC TCG GGA AGG CTA CTC-3'
H'	5'-/5AmMC6/AAA AAA AAA AAA ACG CAC CGC AGT TTG GTC AAT-3'
I'	5'-/5AmMC6/AAA AAA AAA AAA ATC CGA CGC AAC AAT AGG GCA-3'
J'	5'-/5AmMC6/AAA AAA AAA AAA ACC TGC TCG ACA ACT AGA AGA-3'
K'	5'-/5AmMC6/AAA AAA AAA AAA ACC GCG ACC AGA ATT AGA TTA-3'
L'	5'-/5AmMC6/AAA AAA AAA AAA AGC CGA AGC AGA CTT AAT CAC-3'
M'	5'-/5AmMC6/AAA AAA AAA AAA AAC AGG TTC AGA ATC CTC GAC-3'
N'	5'-/5AmMC6/AAA AAA AAA AAA ACT CAT AGA CGA AGC GAG GAC-3'
O'	5'-/5AmMC6/AAA AAA AAA AAA AGT CAC AGA CTA GCC ACG AAG-3'
P'	5'-/5AmMC6/AAA AAA AAA AAA ATG CAT ACA GAC CAA CGG CGA-3'
Q'	5'-/5AmMC6/AAA AAA AAA AAA AAG ACA CGA CAC ACT GGC TTA-3'
S'	5'-/5AmMC6/AAA AAA AAA AAA ATA GAG AGA GTC CAC ACA CGC-3'
U'	5'-/5AmMC6/AAA AAA AAA AAA AGC GTA TCA GCA AGA CCC ATA-3'
Z'	5'-/5AmMC6/AAA AAA AAA AAA AAC CGA TGA CAG TTC ACA GAG-3'
BB'	5'-/5AmMC6/AAA AAA AAA AAA AAT ACG CGA TGG GAT CAG ACT-3'
CC'	5'-/5AmMC6/AAA AAA AAA AAA AGA GCT TCG TGA ACT GAC CTC-3'
HH'	5'-/5AmMC6/AAA AAA AAA AAA ATG ACC CAG ACC AGT TAG TGC-3'

Table S3. List of antibodies and corresponding cDNA-dye conjugate cocktail Ⅰ, Ⅱ and Ⅲ for cycle 1, 2 and 3.

Antibody	Conjugates I	Conjugates Ⅱ	Conjugates Ⅲ
IL-1β	D'-488 (Green)	D'-647 (Red)	D'-Cy3 (Yellow)
IL-8	E'-488 (Green)	E'-647 (Red)	E'-647 (Red)
IL-6	F'-Cy3 (Yellow)	F'-647 (Red)	F'-647 (Red)
VEGF	G'-Cy3 (Yellow)	G'-488 (Green)	G'-488 (Green)
MCP-1	H'-647 (Red)	H'-488 (Green)	H'-488 (Green)
TNFα	I'-647 (Red)	I'-Cy3 (Yellow)	I'-Cy3 (Yellow)
MIF	J'-488 (Green)	J'-Cy3 (Yellow)	J'-Cy3 (Yellow)
GM-CSF	M'-488 (Green)	M'-Cy3 (Yellow)	M'-488 (Green)
IL-2	N'-488 (Green)	N'-647 (Red)	N'-488 (Green)
IL-10	O'-555 (Yellow)	O'-647 (Red)	O'-555 (Yellow)
